# Degradation Network Reconstruction in Uric Acid and Ammonium Amendments in Oil-Degrading Marine Microcosms Guided by Metagenomic Data

**DOI:** 10.3389/fmicb.2015.01270

**Published:** 2015-11-24

**Authors:** Rafael Bargiela, Christoph Gertler, Mirko Magagnini, Francesca Mapelli, Jianwei Chen, Daniele Daffonchio, Peter N. Golyshin, Manuel Ferrer

**Affiliations:** ^1^Systems Biotechnology, Department of Biocatalysis, Institute of Catalysis, Consejo Superior de Investigaciones CientíficasMadrid, Spain; ^2^School of Biological Sciences, Bangor UniversityBangor, UK; ^3^EcoTechSystems Ltd., AnconaItaly; ^4^Department of Food, Environmental and Nutritional Sciences, University of MilanMilan, Italy; ^5^Beijing Genomics InstituteShenzhen, China; ^6^Biological and Environmental Science and Engineering Division, King Abdullah University of Science and TechnologyThuwal, Saudi Arabia

**Keywords:** ammonium, biostimulation, crude oil degradation, enrichment, Mediterranean Sea, metagenomics, microcosm, uric acid

## Abstract

Biostimulation with different nitrogen sources is often regarded as a strategy of choice in combating oil spills in marine environments. Such environments are typically depleted in nitrogen, therefore limiting the balanced microbial utilization of carbon-rich petroleum constituents. It is fundamental, yet only scarcely accounted for, to analyze the catabolic consequences of application of biostimulants. Here, we examined such alterations in enrichment microcosms using sediments from chronically crude oil-contaminated marine sediment at Ancona harbor (Italy) amended with natural fertilizer, uric acid (UA), or ammonium (AMM). We applied the web-based AromaDeg resource using as query Illumina HiSeq meta-sequences (UA: 27,893 open reading frames; AMM: 32,180) to identify potential catabolic differences. A total of 45 (for UA) and 65 (AMM) gene sequences encoding key catabolic enzymes matched AromaDeg, and their participation in aromatic degradation reactions could be unambiguously suggested. Genomic signatures for the degradation of aromatics such as 2-chlorobenzoate, indole-3-acetate, biphenyl, gentisate, quinoline and phenanthrene were common for both microcosms. However, those for the degradation of orcinol, ibuprofen, phenylpropionate, homoprotocatechuate and benzene (in UA) and 4-aminobenzene-sulfonate, *p*-cumate, dibenzofuran and phthalate (in AMM), were selectively enriched. Experimental validation was conducted and good agreement with predictions was observed. This suggests certain discrepancies in action of these biostimulants on the genomic content of the initial microbial community for the catabolism of petroleum constituents or aromatics pollutants. In both cases, the emerging microbial communities were phylogenetically highly similar and were composed by very same proteobacterial families. However, examination of taxonomic assignments further revealed different catabolic pathway organization at the organismal level, which should be considered for designing oil spill mitigation strategies in the sea.

## Introduction

Oil pollution still is a global problem (Yakimov et al., 2007; Bargiela et al., 2015). At present, in many sea regions containment and recovery of oil using booms and skimmers is the method of choice for oil spill first responders (Walther, 2014). Especially in the open sea, the use of dispersants in combination with biostimulation and bioaugmentation agents based on non-toxic, natural low cost formulations, is encouraged, although the majority of tests have been performed at lab-scale (Das and Chandran, 2010; Nikolopoulou and Kalogerakis, 2010; Alvarez et al., 2011; Nikolopoulou et al., 2013). In marine systems, the low concentration of nitrogen, phosphorous, and oxygen, together with their low bioavailability are main factors limiting the degradation of carbon-rich hydrophobic compounds (Howarth and Marino, 2006; Venosa et al., 2010; Ly et al., 2014). Attempts have been made to use different nitrogen sources to promote the growth and selection of different microbial strains with greater catabolic capacity for combating oil spills compared to natural attenuation (Teramoto et al., 2009; Venosa et al., 2010). However, crude oil biodegradation requires about 0.04 g of nitrogen per gram of oil (Atlas, 1981) which makes the choice of nitrogen source pivotal for the whole treatment. Recent data highlighted the possible link between N cycling processes and hydrocarbon degradation in marine sediments (Scott et al., 2014). Therefore, it is essential to select appropriated N-containing biostimulants.

The sources of nitrogen for the degradation tests – mostly performed at lab-scale and in minor occasions at field-scale – included nitrate, ammonium (AMM), urea, uric acid (UA), amino acids and the hydrophobic substance lecithin (Garcia-Blanco et al., 2007; Li et al., 2007; Martínez-Pascual et al., 2010; Venosa et al., 2010; Nikolopoulou et al., 2013; Mohseni-Bandpi et al., 2014). Slow-release nitrogen (AMM-based) fertilizers have also been successfully used for growth stimulation in microbial oil remediation (Miyasaka et al., 2006; Teramoto et al., 2009; Reis et al., 2013). However, AMM has been proved ineffective in treatment of real oil spill due to co-precipitation with phosphates in seawater. In a recent study, we have shown that biodegradable natural fertilizers like UA can be used as cost-efficient biostimulant for enhancing bacterial growth in polluted sediments (Gertler et al., 2015). Each nitrogen source has its advantages and disadvantages, yet overall results have shown that the microbial populations were initially different from those found in the absence of biostimulants and that the degradation efficiency generally increased. It is therefore critical to establish how the whole microbial biodegradation network is affected and whether different pollutants are preferentially degraded as a consequence of amendments of biostimulants.

In an early work using the recently developed AromaDeg analysis (Duarte et al., 2014) and a meta-network graphical approach, we reconstructed the catabolic networks associated to microbial communities in a number of chronically polluted sites (Bargiela et al., 2015). The approach focuses on the usage of metagenomic data, which directly leads to a network that included catabolic reactions associated to genes encoding enzymes annotated in the genomes of the community organisms. We found key catabolic variations associated to changes in community structure and environmental constraints (Bargiela et al., 2015). In this work, this approach was applied to draft the catabolic networks of two different enrichment microcosms set up with sediments from chronically crude oil-contaminated marine sediments from Ancona harbor (Italy) and the natural fertilizer UA or AMM as nitrogen sources (Gertler et al., 2015). Ancona harbor is very close to the urban area and hosts a multi-purpose port receiving cruise liners, passenger ferries, commercial liners and fishing boats. A minor part of the related airborne pollutants is due to the vessels calling at the port while the main contribution comes from road traffic and other human activities. Furthermore, sediments in Ancona harbor are heavily contaminated due to its role as a major ferry terminal and industrial port on the Adriatic Sea. We hypothesize that the microbial community shifts previously observed after addition of UA and AMM (Gertler et al., 2015) may have an influence in the selection of certain catabolic pathways. Potential protein-coding genes (≥20 amino acids long) obtained by direct Illumina HiSeq sequencing of DNA material of the corresponding microcosms (Gertler et al., 2015) constituted the input information in our study.

## Materials and Methods

### Study Site, Microcosm Set-up and Sequence Accession Numbers

The starting point of this study were the meta-sequences previously obtained by direct sequencing from two microcosm sets created using sediment samples from the harbor of Ancona (Italy; 43°37′N, 13°30′15″E), as described previously (Gertler et al., 2015). Both microcosm setups were identical in size, composition, incubation, sampling regime and nutrient concentration with exception of the type of nitrogen source applied. Either AMM or UA were supplied in equimolar amounts of nitrogen. Briefly, one-liter Erlenmeyer flasks (duplicates) were filled with 150 g of sand (Sigma–Aldrich, St. Louis, MO, USA), sterilized and spiked with 10 mL of sterile filtered Arabian light crude oil. One gram of sediment from the sampling site was mixed into the oil-spiked sand as the inoculum. Three hundred milliliters of modified ONR7a medium (Dyksterhouse et al., 1995) (omitting AMM chloride and disodium hydrogen phosphate) was added. We added 5 mL of Arabian light crude oil, which based upon average literature values for density and molecular weight equals about 300 mM of C (Wang et al., 2003), 5 mM of NH_4_Cl and 0.5 mM of Na_2_HPO_4_ resulting in a molar N/P ratio of approximately 10:1. For UA treatment microcosm, 0.21 g (1.25 mmol = 5 mmol N) of UA was provided as nitrogen source while the AMM treatment microcosms were each supplied with 2.5 mL of a 2 M AMM chloride solution (5 mmol; pH 7.8). Both treatments also contained 2.5 mL of a 0.2 M disodium hydrogen phosphate solution (0.5 mmol; pH 7.8). Excess amounts of crude oil were added to compensate for the 35% carbon losses due to evaporation of volatile hydrocarbons over the course of the experiment. Including losses due to evaporation, the C/N/P ratio was approximately 400:10:1. Control treatments were set up: (i) a negative control contained only sterile sand and ONR7a; (ii) two further controls contained sand, ONR7a, crude oil and either UA or AMM chloride solution but no sediment sample; and (iii) one control contained oil, sterile sand, ONR7a medium and a sediment sample, but no additional nitrogen source or phosphorus source was provided. No significant growth was detected under tested control conditions. Under the given assay conditions, the utilization of UA as carbon source is minimal, as the amount of carbon introduced by UA into the microcosms was disproportionately low in contrast to the residual carbon in the sediment and the carbon introduced in form of oil. Briefly, we added 300 mmols of carbon in form of oil and only 6.25 mmols of carbon in form of UA. In addition, the molar ratio C/N in the system (between 10:1 and 40:1, depending UA or AMM was added) implies there was excess of carbon in the medium and thus the growth was limited by N.

The resulting microbial communities from microcosms were destructively sampled after 21 days of incubation at 20°C, the isolated DNA subjected to the paired-end sequencing (Illumina HiSeq 2000) at Beijing Genomics Institute (BGI; China), and gene calling performed as described (Gertler et al., 2015). Taxonomic affiliations of potential protein-coding genes were predicted as described previously (Guazzaroni et al., 2013; Bargiela et al., 2015).

The meta-sequences are available at the National Center for Biotechnology Information (NCBI) with the IDs PRJNA222664 [for MGS-ANC(UA)] and PRJNA222663 [for MGS-ANC(AMM)]. The Whole Genome Shotgun projects are also available at DDBJ/EMBL/GenBank under the accession numbers AZIH00000000 [for MGS-ANC(UA)] and AZIK00000000 [for MGS-ANC(AMM)]. All original non-chimeric 16S small subunit rRNA hypervariable tag 454 sequences were archived at the EBI European Read Archive under accession number PRJEB5322. Note that the samples were named based on the code ‘MGS’, which refers to MetaGenome Source, followed by a short name indicating the origin of the sample and the nitrogen source, as follows: MGS-ANC(AMM) (the harbor of Ancona and AMM as nitrogen source); MGS-ANC(UA) (the harbor of Ancona and UA as nitrogen source).

### Biodegradation Network Reconstruction: Scripts and Commands for Graphics

The web-based AromaDeg resource (Duarte et al., 2014) was used for catabolic network reconstruction. AromaDeg is a web-based resource with an up-to-date and manually curated database that includes an associated query system which exploits phylogenomic analysis of the degradation of aromatic compounds. This database addresses systematic errors produced by standard methods of protein function prediction by improving the accuracy of functional classification of key genes, particularly those encoding proteins of aromatic compounds’ degradation. In brief, each query sequence from a genome or metagenome [MGS-ANC(AMM) and MGS-ANC(UA), in this study] that matches a given protein family of AromaDeg is associated with an experimentally validated catabolic enzyme performing an aromatic compound degradation reaction. Individual reactions, and thus the corresponding substrate pollutants and intermediate degradation products, can be linked to reconstruct catabolic networks. We have recently designed an in-house script allowing the automatic reconstruction of such networks in a graphical format, which was used in present work. The script allows visualization and comparison of the abundance levels of genes encoding catabolic enzymes assigned to distinct degradation reactions as well as substrates or intermediates possibly degraded by distinct microbial communities. The complete workflow, including the scripts and commands used for catabolic network reconstruction has recently been reported (Bargiela et al., 2015).

Note that the sequence material used in the present investigation for biodegradation network reconstruction was based upon single biological microcosm replicate to preserve maximum coverage and sequencing depth as well as for other technical reasons, as described previously (Gertler et al., 2015). For each of the metagenome datasets the rarefaction curves of the observed species were estimated to analyze the species sampling coverage, and found that the rarefaction curves indicate closeness to saturation in each of the samples (Gertler et al., 2015). Therefore, with a single run of paired-end Illumina sequencing we determined populations that really represent the actual state of the microbial community in the microcosms and that biases were not introduced due to differences in microbial coverage. Whether or not more replicates may introduce some differences in the present study was not examined. However, because of the low standard deviation in the cultures (also checked for the representativeness of the microcosm by 16S small subunit rRNA hypervariable tag 454 sequences fingerprinting; Gertler et al., 2015) and the fact that sampled 16S rRNA diversity indicated closeness to saturation, we considered that the presented data are valid. Note that experimental validations (see Experimental Validations of Predicted Biodegradation Capacities) were performed in triplicates (with appropriated standard deviations), on the basis of which metagenome-based predictions were confirmed. Therefore, we considered that the differences at the taxonomic, gene content levels and catabolic capacities herein presented are most likely due to actual biological variability and are not random.

### Experimental Validations of Predicted Biodegradation Capacities

The ability of each of the microcosms to grow on pollutants expected to be degraded, was confirmed as follows. First, UA and AMM microcosms (in triplicates) were obtained as described above but omitting Arabian light crude oil; instead, a mix of pollutants containing naphthalene, 2,3-dihydroxybiphenyl, benzene, *p*-cumate, orcinol, 2-chlorobenzoate, phthalate and phenylpropionate, all from Fluka-Aldrich-Sigma Chemical Co. (St. Louis, MO, USA), was added at a final concentration of 2 ppm each. These pollutants were selected on the basis of existing analytical methods to quantify their concentrations (Bargiela et al., 2015). Control cultures without the addition of sediments but with chemicals and cultures plus sediments but without the addition of chemicals were set up.

The extent of degradation in test and control samples was quantified as follows. Briefly, bacterial cells (from 300 ml culture) were separated by centrifugation at 13,000 *g* at room temperature for 10 min. After supernatant separation, bacterial pellet was used for methanol extraction by adding 1.2 mL of cold (-80°C) high-performance liquid chromatography (HPLC)-grade methanol. The samples were then vortex-mixed (for 10 s) and sonicated for 30 s (in a Sonicator^®^ 3000; Misonix) at 15 W in an ice cooler (-20°C). This protocol was repeated twice more with a 5-min storage at -20°C between each cycle, and the final pellet was removed following centrifugation at 12,000 *g* for 10 min at 4°C. Methanol solution was stored at -80°C in 20-mL penicillin vials until they were analyzed by mass spectrometry and different and complementary separation techniques, namely liquid chromatography electrospray ionization quadrupole time-of-flight mass spectrometry (LC-ESI-QTOF-MS) in positive and negative mode, and gas chromatography-mass spectrometry (GC-MS), as described previously (Bargiela et al., 2015). The abundance levels of mass signatures of tested pollutants and key degradation intermediates, namely, salicylate, gentisate, catechol, benzoate and protocatechuate, were used as indicator of the presence of the corresponding enzymes encoded by catabolic genes.

## Results And Discussion

### Bacterial Community Structures in Microcosms

A graphical approach recently described (Bargiela et al., 2015) was applied to draft the catabolic networks of two different oil-degrading marine microcosms. They were obtained from Ancona harbor sediments which were applied in a series of two enrichment microcosms, where AMM or UA were supplied to introduce equivalent amounts of nitrogen. Using partial 16S rRNA gene sequences obtained in the non-assembled Illumina reads through a metagenomic approach, it was firstly found a relatively high degree of similarity in the emerging communities (Gertler et al., 2015). Proteobacteria were the most abundant (AMM: 74.5%; UA: 74.2%, total sequences), in agreement with the fact that this bacterial group is the most abundant in other chronically crude oil-contaminated marine sediments within the Mediterranean Sea (Bargiela et al., 2015). Noticeably, all proteobacterial families were found in both microcosms (for details see **Table 1**). However, differences in the abundance of some community members could be observed on the basis of corresponding read frequency. As an example, the percentage of members of the *Rhodobacteraceae* and *Enterobacteriaceae* was elevated in microcosms supplied with AMM (18.2% AMM vs. 0.8% in UA and 5.6% in AMM vs. 3.2% in UA, correspondingly). Conversely, lower percentages of members of the *Alteromonadaceae* (9.6%/19.2%), *Halomonadaceae* (5.6%/7.8%), *Moraxellaceae* (0.5%/7.9%) and *Flavobacteriaceae* (1.8%/5.7%) could be detected in the AMM-supplemented microcosm in comparison to UA-based microcosms. At a genus level, 55 out of 57 identified proteobacterial taxa were common in both communities. However, enrichments containing AMM were characterized by higher percentages (referred to total reads) of Alphaproteobacteria, such as *Roseovarius* sp. (1.4% in AMM vs. 0.1% in UA), *Ruegeria* spp. (1.1%/0.1%) and *Sulfitobacter* sp. (1.5%/0.1%), and some Gammaproteobacteria such as *Vibrio* sp. (2.4%/1.1%). In stark contrast to this, the UA-based enrichments showed significantly elevated percentages of members of the Firmicutes (7.4% in UA enrichments/4.9% in AMM enrichments) and Gammaproteobacteria, such as *Aeromonas* spp. (1.5%/0.5%) and *Pseudoalteromonas* sp. (1.9%/0.4%). Highly elevated percentages in UA enrichments were observed for the genera *Acinetobacter* (0.9% in UA enrichments/0.1% in AMM enrichments), *Halomonas* (6.1%/4.2%), *Marinobacter* (16.8%/6.3%) and *Psychrobacter* (6.8%/0.2%). A direct comparison of percentages of potentially oil degrading microbial genera in both microcosms showed a higher percentage of *Acinetobacter* sp. (0.9%/0.1%), *Idiomarina* sp. (0.8%/0.3%), *Oleiphilus* sp. (0.2%/0.03%) and *Marinobacter* sp. (16.8%/6.3%) but lower percentages of *Alcanivorax* sp. (3.9%/4.9%) and *Thalassolituus* sp. (0.04/0.8%) in the UA treatments (Gertler et al., 2015).

**Table 1:** Relative abundance of microbial families within the AMM and UA microcosms.

Family or phylum^1^	Relative abundance (%) based on 16S small subunit rRNA data^1^	
	MGS-ANC(AMM)	MGS-ANC(UA)
*Pseudomonadaceae*	15,24	12,98
*Alcanivoraceae*	4,99	3,94
*Halomonadaceae*	5,20	7,80
*Enterobacteriaceae*	5,62	3,15
*Vibrionaceae*	4,01	2,26
*Aeromonadaceae*	0,90	3,11
*Alteromonadaceae*	9,57	19,18
*Chromatiaceae*	1,06	0,85
*Idiomarinaceae*	0,36	0,81
*Legionellaceae*	0,23	0,91
*Methylococcaceae*	0,54	1,05
*Moraxellaceae*	0,45	7,89
*Oceanospirillaceae*	4,26	4,15
*Pseudoalteromonadaceae*	0,40	2,18
*Shewanellaceae*	2,78	2,59
*Rhodobacteraceae*	18,20	0,81
*Comamonadaceae*	1,64	0,54
*Flavobacteriaceae*	1,75	5,69
Actinobacteria	1,81	2,56
Firmicutes	4,86	7,42
Others	16,12	10,29

*Results are based on the analysis of the partial 16S ribosomal RNA (rRNA) gene sequences extracted from non-assembled DNA sequences obtained by paired-end Illumina HiSeq 2000 sequencing.*

*^1^Only lineages with abundance of reads >1% are shown. Data from Gertler et al. (2015).*

### Biodegradation Networks

As we were interested in obtaining networks that emphasized the catabolic differences within both microcosms, we selected a metagenomic approach to query the presumptive degradation capacities associated to both microcosms. The identification depends heavily on gene abundance and, despite the fact that a substantial faction of less abundant DNA in metagenomes remains undiscovered, the identified catabolic genes are assumed to represent the dominant presumptive pathways in each system. A rarefaction curve of the observed species for both samples to analyze species sampling coverage indicated closeness to saturation in each of the two microcosms (Gertler et al., 2015). In combination with the fact that both samples were sequenced to a similar extent (24,752,834 bp for AMM and 19,364,101 bp for UA; Gertler et al., 2015), this suggests that biases during the comparative analysis within the metagenomes were not introduced due to differences in microbial and sequence coverage.

**FIGURE 1:**
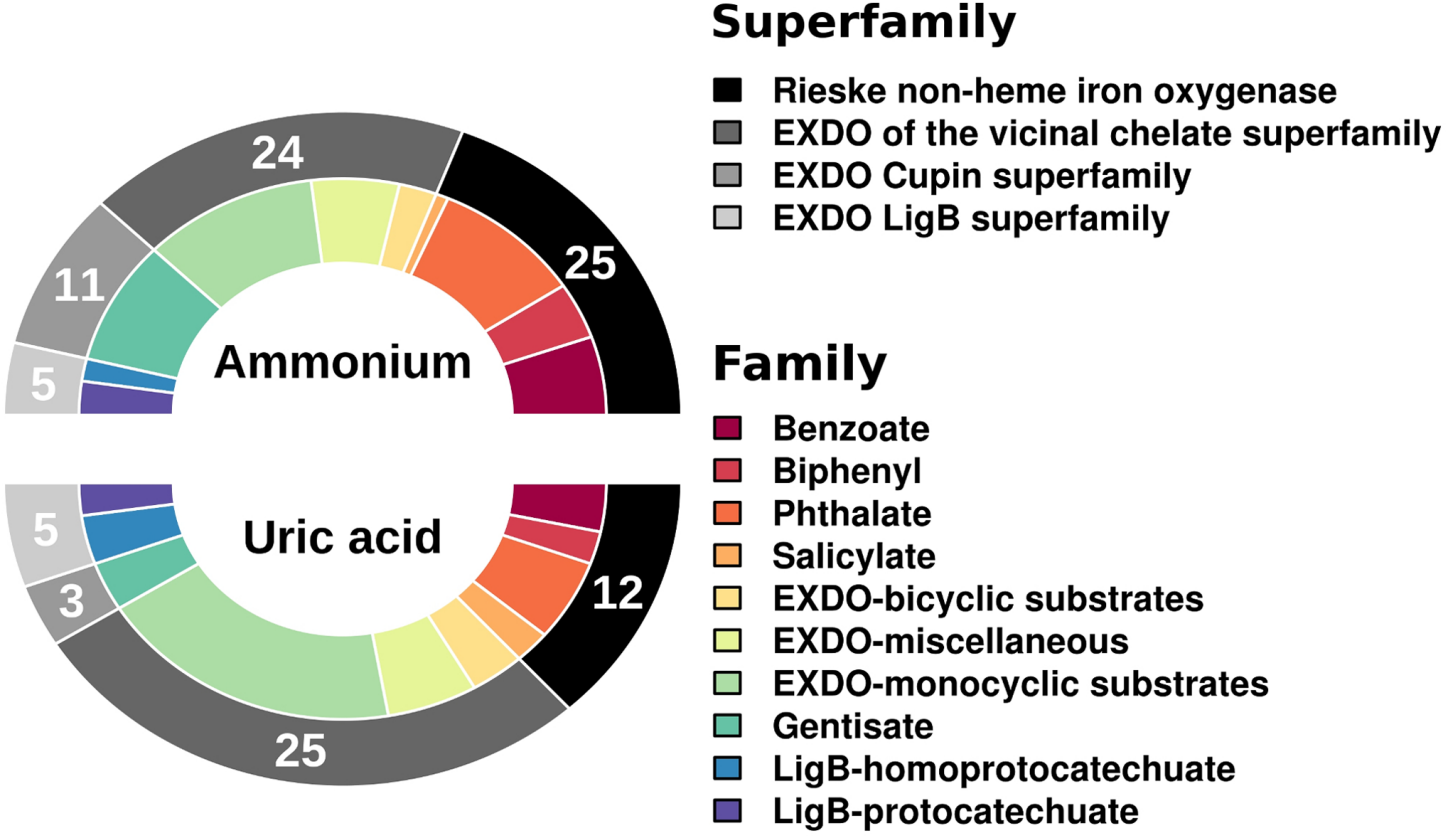
**Number and diversity of sequences of gene families encoding key catabolic enzymes involved in the degradation of aromatic pollutants.** Catabolic genes were identified as follows. Briefly, predicted open reading frames (ORFs) in the metagenomic DNA sequences were filtered by sequence homology (>50%) and minimum alignment length (> 50 amino acids) according to their similarity to the AromaDeg sequences of key aromatic catabolic gene families (and sub-families) involved in the degradation of aromatic pollutants (Duarte et al., 2014). After a manual check, a final list of gene sequences encoding enzymes potentially involved in degradation was prepared.

Using as a query the 27,893 (for UA) and 32,180 (for AMM) potential protein-coding genes (for ≥20 amino acids-long polypeptides) (Gertler et al., 2015), we identified respectively a total of 45 (or 0.16% relative abundance in UA referred to the total number of protein-coding genes) and 65 (or 0.20% relative abundance in AMM) genes encoding catabolic enzymes with matches in AromaDeg (Duarte et al., 2014). This suggests that the biostimulants did not have much influence on the relative abundance of catabolic genes. However, significant differences can be observed when examining the diversity of genes encoding catabolic enzymes assigned to different families (**Figure 1**). The amount of genes encoding Rieske non-heme iron oxygenases and extradiol dioxygenases (EXDO) of the cupin superfamily increased 2- and 4-fold, respectively, and proved more abundant in the AMM microcosm in comparison to those in the UA microcosm (**Figure 1**).

**FIGURE 2:**
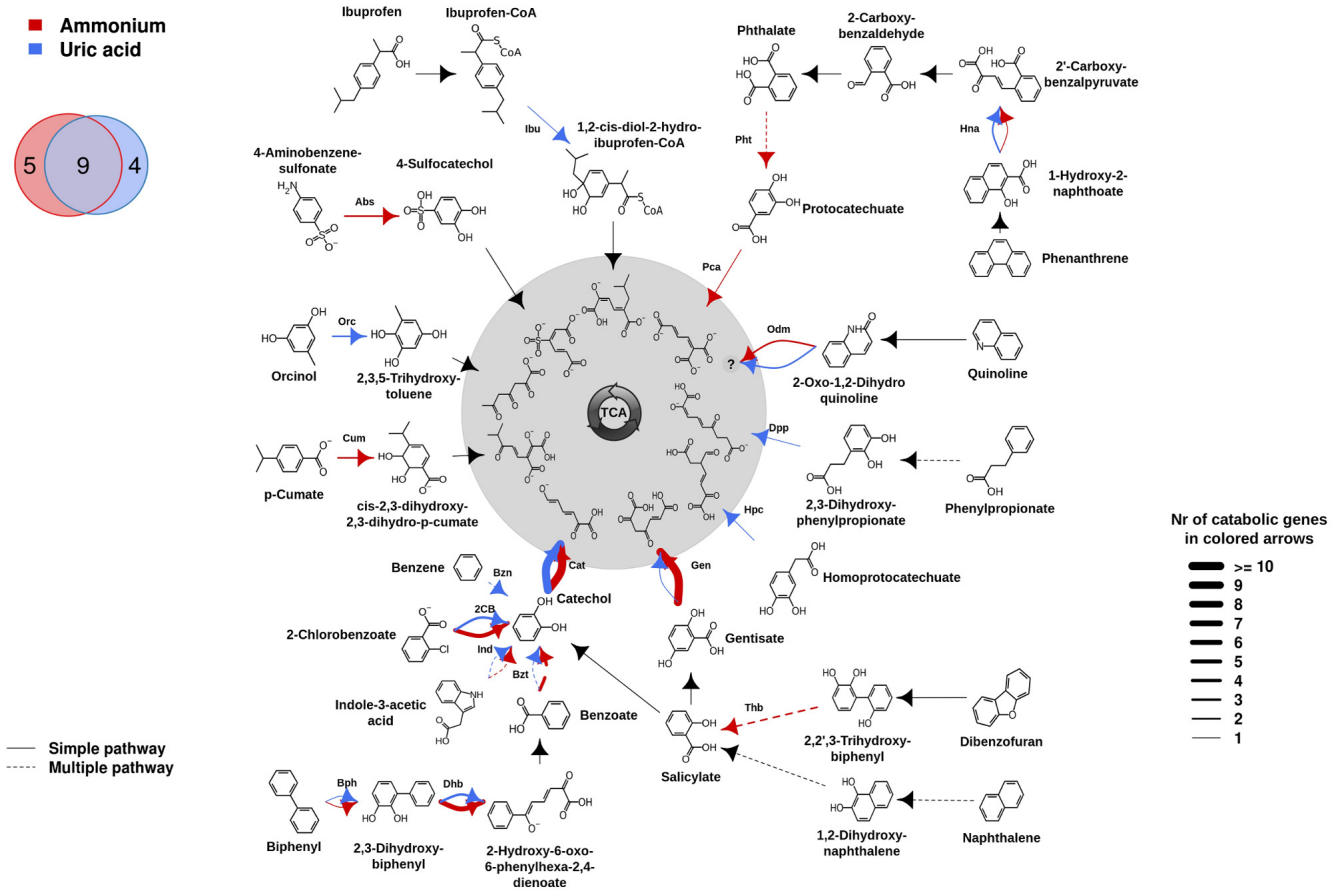
**Potential aromatic catabolic networks in the AMM and UA microcosms (see color code).** The biodegradation network reconstruction was performed as described in “Materials and Methods.” Briefly, catabolic genes were identified as described in the **Figure 1** legend. For network reconstruction, each sequence subsequently was assigned to a metabolic substrate as well as a product (as defined by Duarte et al., 2014) with an assigned code. The putative substrates and products processed in the sample were connected, creating a metabolic network using appropriate scripts and commands (for details, see Bargiela et al., 2015). The number of each catabolic gene assigned to degradation reactions, is represented by the thickness of the lines in the figure and the complete list of substrates possibly degraded by the communities are summarized. Common and microcosm-specific initial pollutants or intermediates for which presumptive degradation signatures were identified are specifically indicated in the Venn diagram. Solid lines represent single step reactions while dotted lines represent degradation steps where multiple reactions are involved (for details see Bargiela et al., 2015). Codes for proteins encoded by genes as follows: Abs, 4-aminobenzenesulfonate 3,4-dioxygenase; Bph, biphenyl dioxygenase; Bzn, benzene dioxygenase; Bzt, benzoate dioxygenase; Cat, catechol 2,3-dioxygenase; 2CB, 2-chlorobenzoate dioxygenase; Cum, *p*-cumate dioxygenase; Dhb, 2,3-Dihydroxybiphenyl dioxygenase; Dpp, 2,3-dihydroxyphenylpropionate dioxygenase; Gen, gentisate dioxygenase; Hna, 1-hydroxy-2-naphthoate dioxygenase; Hpc, homoprotocatechuate 2,3-dioxygenase; Ibu, ibuprofen-CoA dioxygenase; Ind, Rieske oxygenase involved in indole acetic acid degradation; Odm, 2-oxo-1,2-dihydroxyquinoline monooxygenase; Orc, orcinol hydroxylase; Pca, protocatechuate 3,4-dioxygenase; Pht, phthalate 4,5-dioxygenase; Thb, 2,2′,3-trihydroxybiphenyl dioxygenase.

The differences in family shifts may have an influence on degradation capacities provided by microorganisms in AMM and UA microcosms. To assess this, the presumptive aromatic degradation reactions and the substrate pollutants or intermediates possibly degraded by each of the two communities were predicted, and the corresponding degradation networks constructed (**Figure 2**). For that we used the AromaDeg web system that allows identifying catabolic genes and appropriated scripts and commands for graphics (for details see Biodegradation Network Reconstruction: Scripts and Commands for Graphics). Unambiguous reaction specificities could be detected for 35 (in UA) and 48 (in AMM) catabolic genes and were considered in the degradation network (**Figure 2**). However, no clear specificities could be assigned to 4 (in UA) and 11 (in AMM) Rieske oxygenases and 12 (six in UA and six in AMM) EXDO, which subsequently were not considered in the network. As shown in **Figure 2**, on the basis of the presence of genes encoding catabolic genes involved in particular transformations, the potential degradation of nine intermediates involved in the degradation of six key pollutants (2-chlorobenzoate, indole-3-acetate, biphenyl, gentisate, quinoline and phenanthrene) was found to be common for both microcosms. They include the transformation of biphenyl by Bph, 2,3-dihydroxybiphenyl by Dhb, benzoate by Bzt, indole-3-acetate by Ind, catechol by Cat, gentisate by Gen, 2-oxo-1,2-dihydroquinoline by Odm, 1-hydroxy-2-naphthoate by Hna, and 2-chlorobenzoate by 2-chlorobenzoate dioxygenase (2CB). Within them, genes encoding Cat were most abundant in both communities (UA: 17; AMM: 14), in agreement with the fact that catechol is the central intermediate for most cyclic aerobic hydrocarbons degradation (Pérez-Pantoja et al., 2009; Vilchez-Vargas et al., 2013). Gentisate and benzoate/2-chlorobenzoate may be most likely preferentially degraded by microorganisms in the AMM microcosm (10 Gen and 4 Bzt/2CB) in comparison to the UA microcosm (1 Gen and 1 Bzt/2CB). Genomic signatures for the degradation of orcinol (or 3,5-dihydroxytoluene) by Orc, phenylpropionate by Dpp, homoprotocatechuate by Hpc, and benzene by Bzn, were only found in the UA microcosm. The potential degradation of ibuprofen by Ibu, although not being a constituent of the crude oil but possibly originated from bilge water from the cruise lines or urban run-off, was also identified in UA microcosm. In stark contrast, the degradation of 4-aminobenzene-sulfonate by Abs, *p*-cumate by Cum, dibenzofuran by Thb, phthalate by Pht and protocatechuate by Pca, was characteristic for the AMM microcosm.

Note that within all pollutants predicted as being potentially degraded by bacteria inhibiting Ancona port (**Figure 2**), independently whether they are enriched with AMM or UA, only the potential degradation of ibuprofen and 4-aminobenzene-sulfonate was not found associated to bacteria from other chronically crude oil-contaminated sites in oil-polluted sites along the coastlines of the Mediterranean Sea (Bargiela et al., 2015). This suggests that the pollution type and pollutant diversity in Ancona port, which receives chemicals such as alkyl benzene sulfonate detergents and drugs coming from human activities (Martínez-Pascual et al., 2010; Paíga et al., 2013), may have supported the presence of ibuprofen- and sulfonate benzene-growing bacteria. Such bacteria may be further stimulated by either the addition of UA or AMM, respectively.

### Experimental Analysis of Catabolic Capacities in AMM and UA Microcosms

Experimental validation assays were conducted to prove the extent of agreement with metagenomic-based predictions. For that, AMM and UA enrichment cultures were set up in triplicates as described in Section “Experimental Validations of Predicted Biodegradation Capacities,” in which instead of Arabian light crude oil as the carbon source (used for the initial microcosms), naphthalene, 2,3-dihydroxybiphenyl, benzene, *p*-cumate, orcinol, 2-chlorobenzoate, phthalate and phenylpropionate (2 ppm each) were used. The capacity to degrade other pollutants predicted as potential substrates such as ibuprofen, phenanthrene, dibenzofuran, indole-3-acetic acid, 4-aminobenzene-sulfonate and quinoline, could not be experimentally proved because no analytical procedures could be designed for their analysis in the pollutant mix.

**FIGURE 3:**
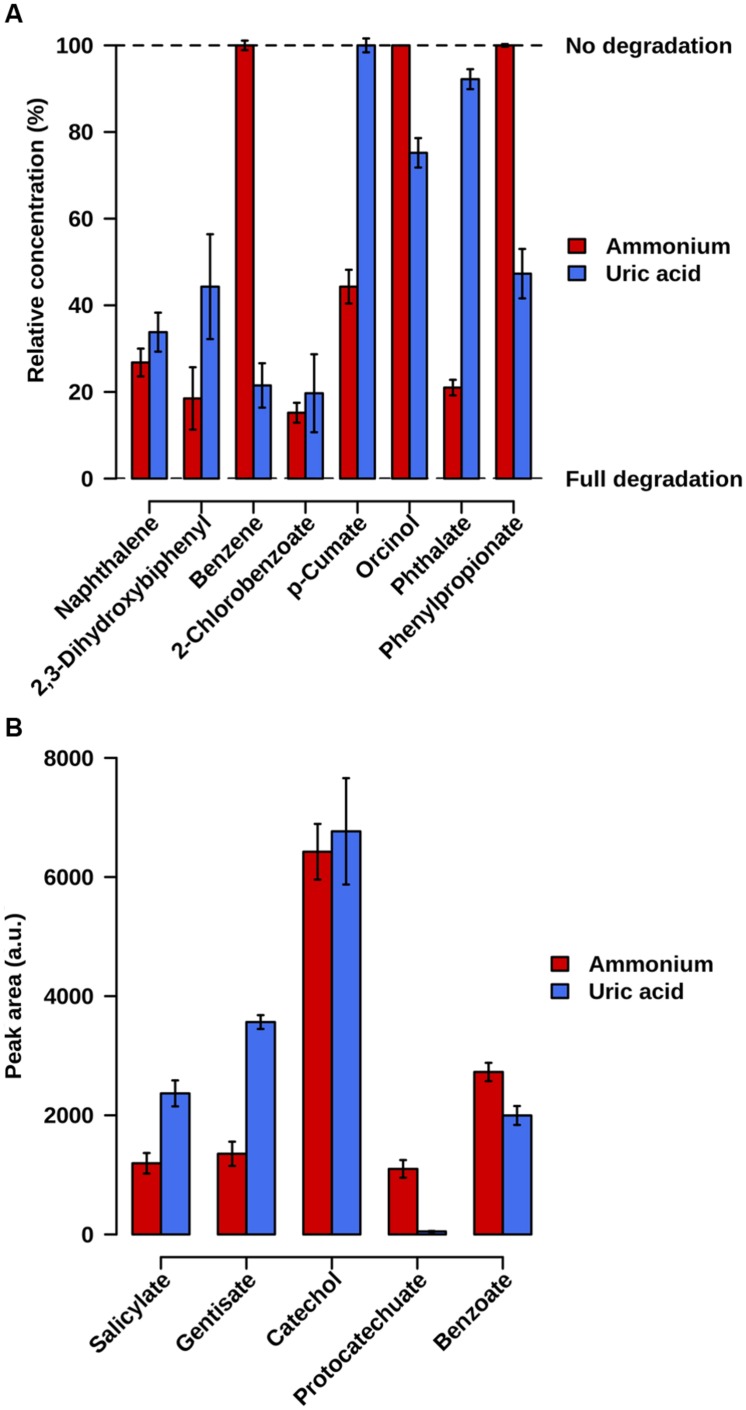
**Relative abundance level of initial substrate pollutants (A) and key chemical intermediates (B), in AMM and UA microcosms containing naphthalene, 2,3-dihydroxybiphenyl, benzene, *p*-cumate, orcinol, 2-chlorobenzoate, phthalate and phenylpropionate (2 ppm each) as carbon source. (A)** The remaining relative concentration of the initial pollutants used to set up enrichment cultures is shown; 100%, no degradation of initial substrate pollutant; 0%, total degradation (absence of pollutant). **(B)** Values represent the peak area of degradation intermediates in arbitrary units (a.u.). The values were calculated, in triplicate microcosms, by comparing the presence and abundance level after 21-days of the microcosm at 20°C experiment compared to the initial point and after considering the controls assays. Standard deviations (SD) are shown.

Samples were taken at 21 days of incubation at 20°C. Fingerprinting by LC-ESI-QTOF-MS and GC-MS was used to confirm the degradation of the initial substrates as well as the existence of degradation intermediates in both cultures. A careful inspection of the mass signatures confirmed the lowering in the abundance level of naphthalene, 2,3-dihydroxybiphenyl, and 2-chlorobenzoate, and the presence of catechol, salicylate, gentisate, and benzoate in both microcosms (**Figure 3**). This demonstrates that the naphthalene-to-salicylate-to-gentisate, 2,3-dihydroxybiphenyl-to-benzoate-to-catechol, and 2-chlorobenzoate-to-catechol degradation pathways occurred or were active in both microcosms. Note that the lower abundance level of gentisate in AMM microcosm may correlate with the 10-fold overabundance of genes encoding Gen enzymes in AMM as compared to UA; this may decrease the pool of gentisate in the microcosm when growing in naphthalene. We further found a decreased level of *p*-cumate only associated to the AMM enrichment. Phthalate degradation mostly associated to the AMM microcosm, as confirmed by the higher extend of phthalate degradation by meaning of its residual percentage at the end of the assay (21 ± 1.8% in AMM vs. 92.2 ± 2.3% in UA) and the 22.2-fold higher abundance of protocatechuate in AMM compared to UA assays. In addition, decreased level of orcinol, benzene and phenylpropionate associated only to UA enrichments (**Figure 3**). Accordingly, the benzene-to-catechol, orcinol-, and phenylpropionate-degradation pathways occurred or were active in the UA microcosm, while *p*-cumate degradation mostly occurred in the AMM enrichments.

The identification of degrading capacities on microcosms depends heavily on enrichment conditions (including cultivation time frame) and bacteria and protein abundance. While these drawbacks are known, the experimental data presented above (**Figure 3**) fully confirmed our sequence-based predictions (**Figure 2**) for the degradation of all eight pollutants tested in each of the two amendments. This suggests that the differences herein predicted in UA and AMM microcosms (**Figure 2**) are due to real biological differences and not random. Uncertainty remains only for phthalate degradation in UA microcosm: experimental analysis demonstrated the slight degradation of this chemical (**Figure 3**), which was not predicted by sequence analysis (**Figure 2**).

### Phylogenetic Identities of Catabolic Genes

We further attempted to analyze the contributions of particular sets of microbes to the entire reconstructed catabolic network, where multiple proteins from multiple organisms may contribute to organic pollutants’ decomposition.

**FIGURE 4:**
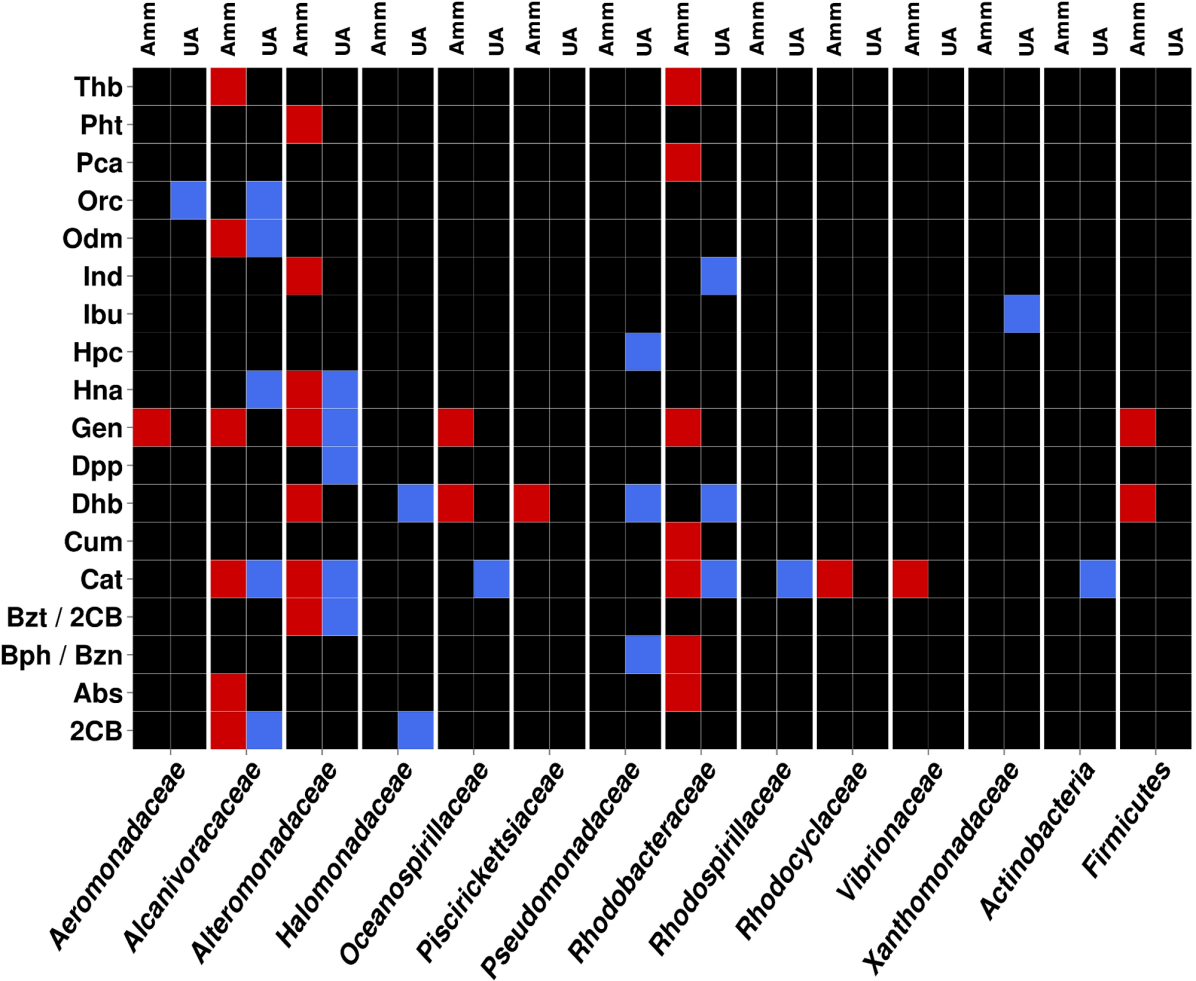
**Heat map showing the contribution of the most relevant bacterial members of AMM and UA microcosm to the degradation network in **Figure 2**.** Contributions of each of the distinct members with unambiguous taxonomic assignation per each of the catabolic gene classes found to constitute the AMM and UA communities are differentiated by a color code. The color indicates the presence of a catabolic gene independently of the abundance level. Gene names/codes are identical to those presented in **Figure 2**.

As the community structure of the two enrichment cultures was well-characterized (Gertler et al., 2015), the taxonomic affiliations of the catabolic genes identified could be unambiguously established at the family and phylum level. For that, we used tools recently published that provide a high level of confidence (Guazzaroni et al., 2013; Bargiela et al., 2015). **Figure 4** shows the contribution of members assigned to the different bacterial families and phyla in both microcosms to pollutant degradation. They included populations closely related to members of *Aeromonadaceae, Alcanivoracaceae, Alteromonadaceae, Halomonadaceae, Oceanospirillaceae, Piscirickettsiaceae, Pseudomonadaceae, Rhodobacteraceae, Vibrionaceae*, and *Xhantomonadaceae*, as well as to a lesser extent for the phyla Actinobacteria and Firmicutes. These comprise bacterial groups well known for their oil biodegrading capabilities (Yakimov et al., 2007; Jin et al., 2012; Guazzaroni et al., 2013). A further careful examination of the data presented in **Figure 4** clearly leads to the occurrence of a different pathway organization at organism level for the catabolism of 18 different pollutants predicted to be degraded.

**FIGURE 5:**
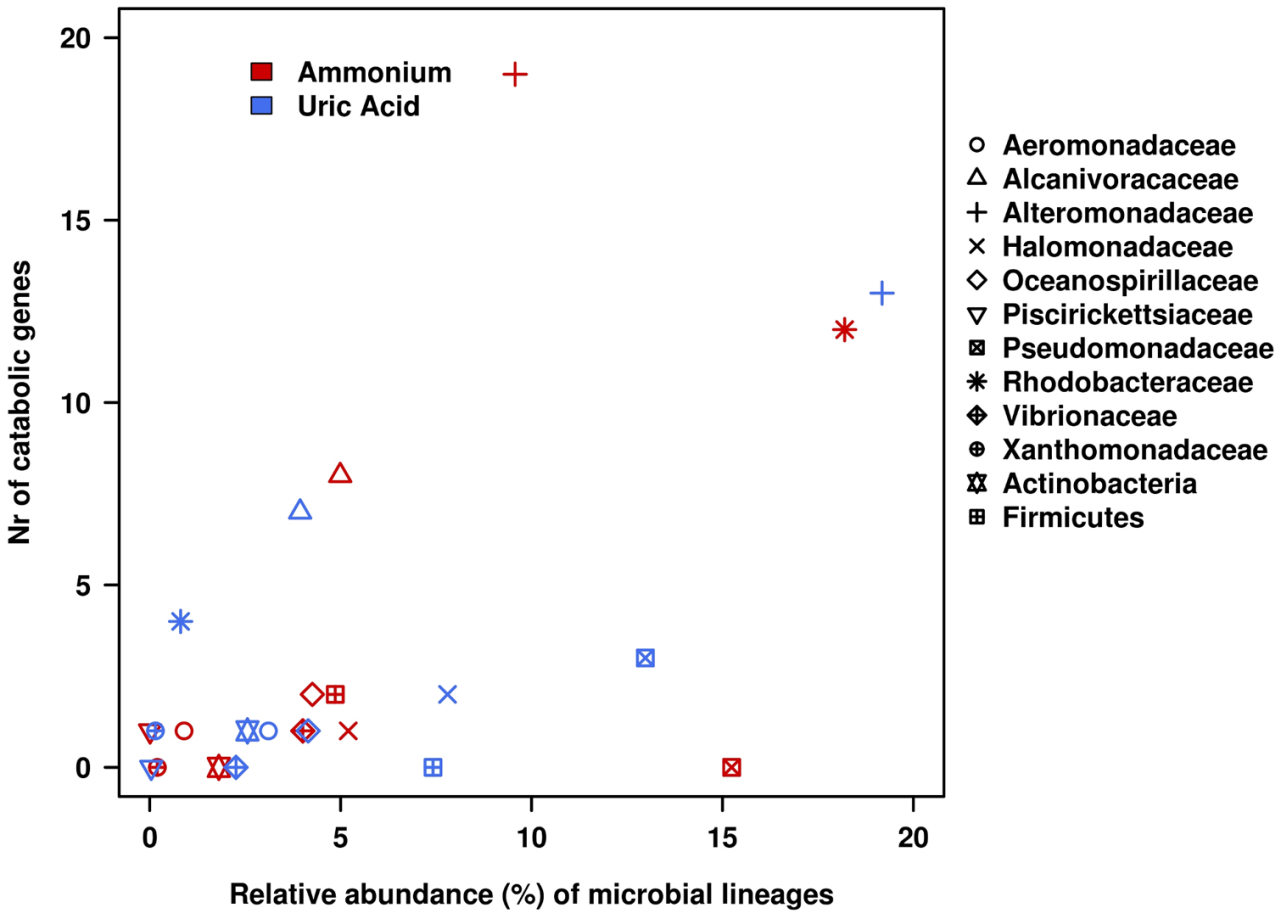
**Relative contributions of bacterial lineages in terms of catabolic genes and 16S rRNA gene within the AMM and UA microcosms.** Catabolic genes were identified by using the web-based AromaDeg resource (Duarte et al., 2014) and their taxonomic assignation at the level of the family or higher was performed as reported (Guazzaroni et al., 2013; Bargiela et al., 2015). The relative abundance of microbial lineages was based on the analysis of 16S small subunit rRNA partial sequences obtained in the non-assembled Illumina reads (Gertler et al., 2015).

As can be seen in **Figure 4**, members of *Alcanivoracaceae, Alteromonadaceae*, and *Rhodobacteraceae* were the major contributors to the networks. They contribute, in combination, to the degradation of 16 out of 18 pollutants predicted in the catabolic network, including dibenzofuran, phenanthrene, indolacetic acid, biphenyl, *p*-cumate, 2-chlorobenzoate, phenylpropionate, aminobenzenesulfonate and gentisate. This is in agreement with the fact that they were among the most abundant members in the established microcosms based on 16S rRNA (**Table 1** and **Figure 5**). Interestingly, *Pseudomonadaceae* which was the second most abundant microbial clade at the level of 16S rRNA in both microcosms (**Table 1** and **Figure 5**), did not contribute to the degradation network in AMM but it does in the UA microcosm (**Figure 4**), where it supports the biphenyl-to-benzoate and homoprotocatechuate degradation.

As shown in **Figure 4**, among the common degradation capacities, a number of observations can be made. First, the degradation of indole acetate by Ind was supported by members of *Alteromonadaceae* in AMM and *Rhodobacteraceae* in UA, which suggests a catabolic replacement. This was also observed for the degradation of biphenyl and benzene (by Bph/Bzn), most likely supported by members of the *Pseudomonadaceae* in UA but *Rhodobacteraceae* in AMM. We identified members of five proteobacterial families (*Aeromonadaceae, Alcanivoracaceae, Alteromonadaceae, Oceanospirillaceae*, and *Rhodobacteraceae*) and of the Firmicutes phylum as key groups for the degradation of gentisate (by Gen) in AMM. By contrast, only members of *Alteromonadaceae* were predicted to support gentisate catabolism in UA. In agreement with this it has been found that AMM promotes the growth of such multiple marine bacteria with the ability to utilize naphthalene (the precursor of gentisate) as a sole carbon in enrichment cultures (Hedlund et al., 1999). Also, the increased abundance of bacteria of the Firmicutes phylum has been demonstrated during bio-stimulation with ammonia (Guazzaroni et al., 2013). The naphthalene-to-gentisate catabolism by bacteria of the family *Alteromonadaceae* has also been found during microcosm assays using seawater and sediment samples obtained after an oil spill along the Korean shoreline without AMM addition (Jin et al., 2012); this agrees with the enrichment of gentisate catabolism by bacteria of this family in UA microcosm.

Multiple bacteria also contributed to the degradation of catechol (by Cat), with members of *Alcanivoracaceae, Alteromonadaceae*, and *Rhodobacteraceae* being common in both treatments. These bacterial groups are known for their capacity to degrade aromatics and haloaromatics to catechol, which can be further catabolised (Brusa et al., 2001; Antunes et al., 2011). Members of the Actinobacteria phyum and *Oceanospirillaceae* family contributed to catechol catabolism exclusively in the UA microcosm, whereas those of *Vibrionaceae* family did so in the AMM treatment. Note that, in accordance with the fact that *cat* genes are the most abundantly present (**Figure 2**) in both microcosms, the number of bacterial groups involved in its catabolism was also the highest (8 in total; **Figure 4**). Therefore, a number of bacterial groups within the microcosms exhibit also partial catabolism redundancy.

Interestingly, we noticed that bacteria from the *Halomonadaceae* family contributed also to degradation of aromatics, particularly, 2-chlorobenzoate (through 2CB) and biphenyl (through Dhb) in the UA microcosm (**Figure 4**). This suggests that halomonads not only participate in the conversion of UA to AMM, which further stimulated growth of hydrocarbonoclastic bacteria (Gertler et al., 2015), but also play specific roles in degradation as herein suggested. This agrees with the fact that bacteria from the genus *Halomonas* are capable of degrading chlorobenzoates (de la Haba et al., 2011) and aromatics compounds such as benzoate and catechol (Piubeli et al., 2012), that are intermediate products of biphenyl and 2-chlorobenzoate degradation.

## Conclusion

Here, we report that different biostimulants applied in chronically polluted sediments have caused significant alteration in degradation capacities, while having no major effect on the taxonomic composition of microbial communities at the level of the family or higher. Experimental validation was conducted for at least eight of the predicted catabolic capacities, and good agreement with metagenomics-based predictions was observed. On the other hand, the metagenomics-guided metabolic reconstruction allowed us to refine the assignment of roles of community members in the utilization of multiple substrates and found different pathway organization at organism level. For example, while biphenyl degradation by Bph, DhB, and Bzt enzymes seems to be carried out by bacteria of *Pseudomonadaceae*, *Halomonadaceae*, and *Rhodobacteraceae* in UA, those of *Alteromonadaceae, Oceanospirillaceae, Picirickettsiaecae*, and Firmicutes may be involved in an alternative pathway in AMM. This demonstrates that different microbial members within microcosms obtained with different nitrogen sources may exhibit partial functional redundancy, and thus, may have a high level of common catabolic capacities. The present investigation provides an estimation of such common and distinct degrading capacities. Indeed, herein we found that 50% of the predicted degradation capacities were common for microorganisms in AMM and UA microcosms (**Figure 2**). However, according to the microbial biodegradation networks herein reconstructed, we also found that the two different biostimulants investigated, UA and AMM, have also changed substrate utilization capacities and preferences, which must be considered for the design of petroleum bioremediation techniques. This was demonstrated by showing that UA enriched for bacteria with the capability of degrading pollutants otherwise not degraded, or possibly degraded at low level, by those stimulated by the addition of AMM, and vice versa.

Therefore, the results of this study show that smart formulations based on the application of multiple nitrogen sources, rather than commonly used single sources (mostly AMM), for example, may increase the efficiency of the biological removal of the widest diversity of aromatic pollutants and could be essential to support effective biodegradation strategies in response to an oil spill incident or in response to chronical pollution. Thus, as herein demonstrated, the utilization of both AMM and UA in conjunction will have a double aim. In one side, AMM may most likely enhance the bio-stimulation of bacterial populations capable of degrading heavy oil components such as naphthalene, phenathrene and dibenzofuran, as well as sulfonated-benzenes and substituted benzoate derivatives such as p-cumate (**Figure 2**). In other side, UA will promote the growth of bacteria most active against benzene, orcinol-, ibuprofen- and phenyl-propionate (**Figure 2**). This will provoke a significant increase in multiple aromatics consumption in polluted areas. Having said that, this work seems to introduce a promising way for future oil-based contamination handling techniques. In this context, it would be very interesting to test the overall cleaning capacity (if any) on a real oil-contaminated marine sample. For that, also another point will be to use the combination of the UA and AMM, which was herein not presented in microcosm assays. It would be interesting to see their combinatory effect in the overall degradation capacity and taxonomic distribution of the microbial niche depending also on their ratio, so to find optimal nitrogen-containing formulations in real scenarios.

We would like to stress the attention to the fact that similarities regarding microbial community composition in the AMM microcosm from Ancona port with those reported in enrichments from surface water bodies at other Mediterranean sites, were found (Gertler et al., 2012). However, a similar comparison with the results from UA microcosm cannot be established because the limited information available. In fact, to the best of our knowledge, there have been only three studies that thoroughly investigated the use of UA in bioremediation trials (Koren et al., 2003; Knezevich et al., 2006; Nikolopoulou et al., 2013). Those studies, however, did not use UA in comparison to other nitrogen sources such as AMM, both in respect to their effect in microcosm population structures and catabolic preferences. Accordingly, herein we reported first evidences linking UA to catabolic preferences at the bacterial level, in comparison to the commonly use nitrogen source AMM.

## Conflict of Interest Statement

The authors declare that the research was conducted in the absence of any commercial or financial relationships that could be construed as a potential conflict of interest.
